# Perceptions of Medical Malpractice Liability and Informed Consent Among Hospital Healthcare Workers: A Cross-Sectional Study

**DOI:** 10.3390/healthcare14172680

**Published:** 2026-08-23

**Authors:** Alina Doina Tănase, Ștefania Dinu, Raluca-Mioara Cosoroabă, Adriana Pădure, Cristina Ioana Talpoș Niculescu

**Affiliations:** 1 Department of Professional Legislation in Dental Medicine, Faculty of Dental Medicine, “Victor Babes” University of Medicine and Pharmacy, Eftimie Murgu Square No. 2, 300041 Timisoara, Romania; tanase.alina@umft.ro (A.D.T.); cosoroaba.raluca@umft.ro (R.-M.C.); 2 Research Centre in Dental Medicine Using Conventional and Alternative Technologies, Faculty of Dental Medicine, “Victor Babes” University of Medicine and Pharmacy, Eftimie Murgu Square No. 2, 300041 Timisoara, Romania; 3 Pediatric Dentistry Research Center (Pedo-Research), Department of Pediatric Dentistry, Faculty of Dental Medicine, “Victor Babes” University of Medicine and Pharmacy, Eftimie Murgu Square No. 2, 300041 Timisoara, Romania; 4 Department of Oro-Dental Diagnosis and Ergonomics, Faculty of Dental Medicine, “Victor Babes” University of Medicine and Pharmacy, Eftimie Murgu Square No. 2, 300041 Timisoara, Romania; adriana.padure@umft.ro (A.P.); ioana.talpos-niculescu@umft.ro (C.I.T.N.)

**Keywords:** medical malpractice, informed consent, defensive medicine, malpractice anxiety, legal literacy, healthcare workers, cross-sectional study

## Abstract

***Background and Objectives:*** Rising malpractice litigation and the strengthening of patient-rights legislation have changed the way hospital staff approach informed consent and legal risk. This study aimed to (i) assess the distribution of informed-consent knowledge, malpractice anxiety, and defensive practice across professional and departmental groups; (ii) identify independent predictors of high defensive practice; and (iii) evaluate experience-related moderation of the legal-literacy–anxiety association and predictor consistency across defensive-practice severity. ***Methods:*** We conducted a cross-sectional survey of 146 healthcare workers (physicians, nurses, midwives, and allied health professionals) from four departments of a tertiary academic hospital network, using a 46-item pilot-tested instrument measuring informed-consent knowledge (0–20), malpractice anxiety (0–100), and defensive-practice behaviors. ***Results:*** Physicians showed higher informed-consent knowledge than nurses (14.1 vs. 12.0 points, *p* < 0.001) but also higher malpractice anxiety (63.4 vs. 57.6, *p* = 0.015). High defensive practice was associated with a prior malpractice claim (adjusted odds ratio 3.24), surgical specialty (2.47), low consent literacy (2.21), physician role (2.10), and knowing a sued colleague (1.88). Anxiety was highest in emergency medicine; predictors also operated across ordered defensive-practice severity, and the legal-literacy–anxiety association was stronger at lower experience (interaction *p* = 0.023). ***Conclusions:*** Knowledge, anxiety, and defensive practice were unevenly distributed; defensive practice reflected professional context and direct or vicarious litigation exposure, while the legal-literacy–anxiety association varied with experience. Medico-legal education may therefore be best paired with structured consent procedures and experience-sensitive support; larger multi-center studies are needed.

## 1. Introduction

### 1.1. Legal Framework Governing Medical Malpractice and Informed Consent

Medical malpractice liability and informed-consent obligations are regulated throughout Europe, but the applicable frameworks are heterogeneous and may combine statutory provisions, case law, professional rules, and administrative instruments. The Convention on Human Rights and Biomedicine provides a common regional baseline for free and informed consent and was ratified by Romania in 2001 [1]. Throughout this article, medical malpractice liability denotes the civil liability of healthcare personnel and of healthcare providers for professional error committed in the exercise of the medical act, in the sense given to that term by the legislation applicable to the study setting; it does not refer to disciplinary or criminal liability, which follow separate procedures and are outside the scope of this study. In Romania, the jurisdiction of the present study, this liability is regulated primarily by Title XVI of Law no. 95/2006 [2] on healthcare reform. Article 653(1)(b) defines malpractice as the professional error committed in the exercise of the medical or medico-pharmaceutical act that generates prejudice to the patient and entails the civil liability of medical personnel and of the provider of medical, sanitary and pharmaceutical products and services [2]. The same title delimits the conduct capable of engaging this liability: Article 653(2) covers professional error, including error resulting from exceeding the limits of one’s competence; Article 654 covers negligence, imprudence and insufficient medical knowledge; and Article 655 covers prejudice arising from breaches of professional confidentiality, from failure to obtain informed consent, and from failure to provide medical assistance [2]. The term “malpractice” is used in this article in this circumscribed statutory sense and not as a general synonym for adverse outcome, complication, medical error, or professional misconduct. Accordingly, failures related to informed consent may fall within malpractice liability under Romanian law, but the constructs are not synonymous in this study: malpractice liability denotes the broader civil-liability framework and respondents’ perceived exposure to it, whereas informed consent is a specific legal and ethical duty assessed as a separate knowledge domain. Both terms are retained in the title to reflect this nested but analytically distinct relationship.

Importantly, the occurrence of a professional error is not by itself sufficient to engage liability. Under Articles 653–655 of Law no. 95/2006, read together with the general rules of civil liability of the Romanian Civil Code, four conditions must be established cumulatively: an illicit act consisting of a professional error, prejudice sustained by the patient, a causal link between the error and the prejudice, and fault on the part of the healthcare professional [2]. A diagnostic or therapeutic decision, or a deficiency in the information provided to the patient, is therefore actionable only where these conditions are met, and not merely because an unfavorable outcome has occurred. The legal threshold therefore differs conceptually from clinicians’ perceived liability risk. Because this study examines perceptions rather than adjudicated liability, it focuses on perceived medico-legal risk as a potential correlate of clinical behavior; the empirical evidence supporting this association is summarized below.

The normative model governing the physician–patient relationship has meanwhile moved from paternalism toward patient autonomy, shared decision-making and documented disclosure. The conceptual foundations of this shift were established in the bioethics literature of the 1980s and are set out in the standard framework of biomedical ethics [3,4]; its consolidation in positive law has continued into the early twenty-first century, with several European and common-law jurisdictions moving from physician-centered disclosure standards toward patient-centered standards and increasingly treating consent as a documented dialogue rather than a signature [1,5,6]. Litigation exposure is meanwhile substantial and unevenly distributed. In the United States, whose liability system has been described in detail [7], a national analysis of paid malpractice claims between 1992 and 2014 found that the overall rate of paid claims fell by roughly half over the period, while marked and persistent differences between specialties remained, with surgical and other procedural specialties consistently at the top of the distribution [8]. In Europe, liability regimes, insurance arrangements, and consent norms differ substantially from the US model. Nevertheless, European review literature identifies defensive medicine across diverse national settings [9,10], and qualitative work among Danish general practitioners describes how concerns about complaints and medico-legal consequences enter clinical decision-making [11]. These observations are relevant to the present study because they support examining perceived liability concerns as a correlate of clinical conduct without assuming that perceived risk is more influential than the governing legal standard.

Informed consent occupies a uniquely visible position within this legal landscape because it is simultaneously an ethical obligation, a legal requirement, and a routine clinical task [12,13]. In the Romanian legal framework, informed consent is governed by complementary statutory, regulatory, and professional instruments. Law no. 46/2003 on patients’ rights establishes, in Chapter II (Articles 4–12), the patient’s right to information on the available medical services, on the identity and professional status of the providers, and on the proposed diagnostic and therapeutic methods, their potential risks, the available alternatives, the consequences of not undergoing treatment, and the diagnosis and prognosis, conveyed in respectful, clear and accessible language; Chapter III (Articles 13–20) governs consent to a medical intervention, including the patient’s right to refuse or to interrupt an intervention while assuming responsibility in writing (Article 13) and the arrangements applicable where consent cannot be obtained (Articles 14–15) [14]. Law no. 95/2006 states the corresponding obligation on the professional side: Articles 660–664 of Title XVI require that, before methods of prevention, diagnosis or treatment carrying a potential risk for the patient are applied, the physician explain them to the patient and obtain the patient’s written consent, and they regulate consent in emergencies and for patients lacking discernment [2]. The norms approved by Order of the Minister of Health no. 1.410/2016 further specify the content and the documentation of this information duty [15]. The current Code of Medical Deontology of the Romanian College of Physicians, adopted by Decision no. 2/2025 and effective from 1 January 2026, reinforces these duties in Chapter IV (Articles 25–29), which addresses granting and withdrawal of consent, consent for minors and adults who lack capacity, the physician’s duty to provide relevant information in a form adapted to the patient’s understanding, and consent in emergency situations [15].

These obligations are not distributed uniformly across the care team. Under Article 660 of Law no. 95/2006, the duty to explain an intervention carrying potential risk and to obtain and document the patient’s written consent rests with the physician, who bears the corresponding legal responsibility [2]. Nurses and midwives do not, as a rule, obtain consent on behalf of the physician for physician-performed interventions; their statutory role, defined by Government Emergency Ordinance no. 144/2008 on the exercise of the professions of generalist nurse, midwife and medical assistant, is to inform patients about, and to obtain consent for, the care acts that fall within their own professional competence—for midwives including the autonomous surveillance and assistance of physiological pregnancy and birth—and otherwise to contribute to the information, verification, witnessing and documentation of the consent process [16]. Consent is therefore treated here as a legally regulated act for which responsibility is graded across professional roles, which is precisely why consent-related knowledge and consent-related anxiety were measured separately in physicians, nurses, midwives and allied health professionals rather than assumed to be common to them.

Although the formal elements of valid consent—disclosure, comprehension, voluntariness, and capacity—are well established in legal and ethical standards, considerable variation exists in how clinicians understand and operationalize them [17]. Surveys of clinicians repeatedly demonstrate gaps between self-reported confidence in obtaining consent and objective knowledge of its legal components [18], and more recent empirical work documents substantial shortfalls in the consent process itself: a 2021 systematic review of empirical studies of consent comprehension reported that patients’ understanding of the information disclosed to them is frequently incomplete, with recall of disclosed risks below 50% in a large proportion of the included studies [19], while a 2020 systematic review of qualitative evidence on surgical consent found that clinicians commonly experience the consent encounter as a time-pressured documentation requirement rather than as a decision-making dialogue [20]. These gaps matter because failure to obtain informed consent is an expressly actionable basis of civil liability under Article 655 of Law no. 95/2006 [2] and a recurrent allegation category in claims arising from procedural specialties [21], and because perceived legal vulnerability can distort clinical communication.

### 1.2. Defensive Medicine and Its Implications for Clinical Practice

A particularly consequential downstream effect of malpractice perception is defensive medicine, defined as the ordering of tests, procedures, or referrals—or the avoidance of high-risk patients and interventions—primarily to reduce perceived legal exposure rather than to advance patient welfare [22]. In concrete clinical terms, defensive medicine is conventionally divided in the empirical literature into two forms—a distinction introduced by survey research on high-risk specialties and confirmed by a 2022 systematic review of how the concept is defined in the European medical literature [10,23]: assurance behaviors, such as ordering additional imaging or laboratory tests of marginal utility, requesting supplementary specialist consultations, or documenting in excessive detail “for the file”; and avoidance behaviors, such as declining to care for complex or litigation-prone patients or avoiding high-risk procedures altogether. Defensive practice inflates healthcare costs, exposes patients to unnecessary risk, and erodes professional satisfaction [9,24]. While defensive medicine has been extensively documented among physicians, far less is known about how it manifests across the broader healthcare workforce, including nurses, midwives, and allied health professionals who also participate in consent and disclosure [25].

The psychological dimension of liability perception is frequently overlooked in favor of behavioral or economic measures. Malpractice anxiety—the affective burden associated with the fear of litigation—may operate independently of objective legal knowledge and may be more strongly tied to vicarious experiences, such as knowing a sued colleague, than to direct exposure [26]. Treating anxiety as a measurable construct, rather than an incidental byproduct, allows investigators to disentangle the cognitive and emotional drivers of defensive behavior, which may require fundamentally different interventions [27].

Importantly, healthcare workers are not a homogeneous group. Profession, specialty, years of experience, and institutional setting plausibly interact in shaping liability perceptions [28]. A senior surgeon and a junior nurse may share the same workplace yet inhabit very different medico-legal realities. Few studies have examined these interactions explicitly, and fewer still have applied moderation or interaction modeling to determine whether, for example, the protective effect of legal knowledge on anxiety depends on professional seniority. Such interaction effects, if present, would have direct implications for how educational interventions are targeted.

### 1.3. Research Problem, Research Questions, and Hypotheses

Research problem. Hospital care is delivered by mixed professional teams that operate under a single medico-legal framework while occupying different positions within it. Within such teams, it remains unclear how informed-consent knowledge (legal literacy), malpractice anxiety, and defensive practice are distributed across professional and departmental groups, which of these factors is most closely associated with defensive behavior, and whether the relationship between legal knowledge and legal fear is uniform across the career span. Resolving this uncertainty yields the information that hospital managers, educators, and policymakers need in order to design effective medico-legal training and support.

Conceptual definitions. Throughout this article, legal literacy denotes clinicians’ objective knowledge of the legal requirements of informed consent, operationalized as described in Section 2.3; malpractice anxiety denotes the affective burden associated with the fear of litigation; and defensive practice denotes the assurance and avoidance behaviors defined above. Medical malpractice liability is used in the statutory sense set out at the beginning of this section.

Focus of the study. The study focuses on the perceptions of hospital healthcare workers—not on litigation outcomes, claim files, or patient-reported experience—and on the association of those perceptions with self-reported defensive behavior. Its aim was to quantify informed-consent knowledge, malpractice anxiety, and defensive practice in a mixed hospital workforce and to determine which individual and contextual characteristics are associated with defensive practice.

Research questions. The present cross-sectional study addressed this problem within a single tertiary-care institutional network by answering three research questions. RQ1 (Objective 1): How are informed-consent knowledge, malpractice anxiety, and defensive practice distributed across professional and departmental groups? RQ2 (Objective 2): which characteristics—professional role, surgical specialty, consent literacy, and direct or vicarious litigation exposure—independently predict high defensive practice? RQ3 (Objective 3): does clinical experience moderate the relationship between legal literacy and malpractice anxiety, and do the identified predictors operate consistently across the whole severity range of defensive practice?

Hypotheses. Four hypotheses were specified a priori on the basis of the literature reviewed above. H1: informed-consent knowledge is higher in physicians than in nurses, reflecting the physician’s primary legal responsibility for obtaining consent [2,16]. H2: malpractice anxiety is higher in time-pressured, procedurally intensive departments (emergency medicine, surgery, obstetrics) than in internal medicine [23,28]. H3: high defensive practice is independently associated with direct and vicarious litigation exposure and with low consent literacy, after adjustment for professional role and specialty [25,26]. H4: the association between legal literacy and malpractice anxiety is moderated by clinical experience, being stronger at lower levels of clinical experience and attenuated at higher levels of experience [26,27]. Hypotheses H1–H3 are directional; H4 is the pre-specified interaction hypothesis, and all four were tested with the analyses described in Section 2.4. Section 3 is organized to follow the three objectives.

## 2. Materials and Methods

### 2.1. Study Design and Setting

This was a single-network, cross-sectional, questionnaire-based study conducted between February 2026 and April 2026 across the affiliated hospitals of a tertiary academic medical center. The institutional network comprised one emergency clinical county hospital and three departmental units spanning surgery, internal medicine, emergency medicine, and obstetrics and gynecology. The cross-sectional design was selected because the primary aim was to describe the distribution and structure of perceptions at a defined point in time, rather than to establish temporal or causal relationships. The reporting of this study follows the STROBE guidelines for observational cross-sectional research.

The study population consisted of clinically active healthcare workers, including resident and attending physicians, registered nurses, midwives, and allied health professionals who participate in patient care and consent-related activities. For the purposes of this study, “allied health professionals” comprised clinically active non-physician, non-nursing staff who contribute to patient care and to the preparation or witnessing of consent-related documentation, namely physiotherapists, radiology technicians, and laboratory technicians with direct patient contact. Administrative staff without direct patient contact were excluded. The network employed approximately 520 eligible healthcare workers during the study window, and the sampling frame was constructed from departmental personnel rosters. Each participating department designated a local coordinator who facilitated distribution and ensured that recruitment did not interfere with clinical duties. The study was conducted in accordance with the Declaration of Helsinki and approved by the Ethics Committee of “Victor Babeș” University of Medicine and Pharmacy Timisoara (No. 45/26 January 2026).

### 2.2. Participants and Sampling

We employed stratified proportional sampling across the four target departments to ensure that the final sample reflected the professional composition of the network. Within each stratum, the target number of participants was set proportionally to the size of the departmental workforce, and eligible workers were then selected at random from the departmental roster and invited to participate; randomly selected non-respondents were not replaced by convenience sampling, and recruitment within a stratum ended when the proportional allocation was reached. This procedure corresponds to stratified random sampling with proportional allocation rather than quota filling; nevertheless, as in any voluntary survey, differential willingness to participate cannot be fully excluded and is acknowledged as a potential source of selection bias in the Limitations section (Section 4.2). The target sample size was determined a priori using an anticipated proportion of high defensive practice of 35%, a 7% absolute precision, a 95% confidence level, and a finite-population correction for the eligible workforce of approximately 520, yielding a minimum of 133 participants; this was inflated by approximately 10% to accommodate incomplete responses, giving a recruitment target of 146.

Of 170 healthcare workers approached, 146 returned analyzable questionnaires, corresponding to a response rate of 85.9%. Inclusion criteria were a minimum of six months of clinical experience within the network and current involvement in direct patient care. Exclusion criteria comprised long-term leave during the study window, refusal to consent, and questionnaires with more than 15% missing items. Participants were assured of anonymity, and no personally identifying information was collected; a randomly generated alphanumeric code linked the responses for quality-control de-duplication only. No financial incentive was offered, minimizing the risk of compliance bias.

### 2.3. Instrument and Variables

Data were collected using a structured self-administered instrument developed by the research team and refined through expert review and a pilot phase involving 28 healthcare workers; pilot participants were excluded from the final analysis in order to avoid contamination of the main sample, and the sampling frame for the main study was reduced accordingly (approximately 492 eligible staff, which remained ample for the recruitment target of 146). The instrument comprised a sociodemographic and professional-characteristics section (not scored), followed by 46 scored items organized into four subscales: (i) an informed-consent knowledge scale of 20 dichotomously scored items (range 0–20) addressing the legal elements of valid consent, capacity assessment, and documentation; (ii) a Malpractice Anxiety Index of 12 items rated on a five-point Likert scale; (iii) a defensive-practice subscale of 10 items; and (iv) a Legal-Literacy Attitudes subscale of 4 items exploring self-assessed adequacy of legal training and attitudes toward medico-legal regulation, used descriptively and in the reliability analyses. The Malpractice Anxiety Index covers four themes: the perceived personal likelihood of being sued, fear of the professional and reputational consequences of a complaint, worry that legal considerations intrude on clinical decision-making, and the emotional burden associated with adverse events and litigation; item scores (1–5) are summed and linearly transformed to a 0–100 metric, with higher values indicating greater anxiety. Because the index aggregates twelve Likert-type items into a summated score, it was treated as an approximately continuous variable in parametric analyses, an approach supported by methodological work on summated rating scales [29,30]; non-parametric (Spearman) coefficients were computed in parallel and yielded the same pattern of results. The defensive-practice subscale asks how frequently the respondent engages in assurance behaviors (e.g., ordering additional tests or consultations primarily for legal protection, over-detailed documentation) and avoidance behaviors (e.g., avoiding high-risk patients or procedures), each item rated on a five-point frequency scale from 1 (never) to 5 (always); item scores are summed to a total between 10 and 50, with higher totals indicating more frequent defensive behavior. For reproducibility, if S denotes the raw 12-item anxiety sum (range 12–60), the 0–100 index was calculated as 100 × (S − 12)/48.

The principal outcome variable for predictive modeling was “high defensive practice,” defined a priori as a defensive-practice total score in the upper tertile of the sample distribution (in this sample, a summed score of 32 or above; the lower and middle tertiles corresponded to scores of 10–24 and 25–31, respectively). Tertile-based categorization was pre-specified to permit comparison with previous defensive-medicine surveys and to provide an interpretable outcome for logistic modeling; because any categorization of a continuous score discards information, all categorical analyses were complemented by analyses that retain the continuous or ordered nature of the scores (the correlation analyses in Section 3.3 and the ordinal regression in Section 3.4). Legal literacy was operationalized both continuously and as a binary indicator (low vs. adequate) using a pre-specified threshold corresponding to the median knowledge score of the sample. Key explanatory variables included profession, medical specialty grouping, years of clinical experience, prior personal malpractice claim, knowledge of a sued colleague, and hospital sector. Instrument reliability was assessed using Cronbach’s α and McDonald’s ω, and construct coherence was examined through item–total correlations, reported among the advanced analyses.

Appendix A provides a consolidated scoring guide for the 46-item study instrument. The verbatim questionnaire and item-level coding key are available from the corresponding author upon reasonable request. No additional participant-level analyses are presented beyond those reported in the main text.

### 2.4. Statistical Analysis

All analyses were performed using IBM SPSS Statistics, version 31.0.2.0 (IBM Corp., Armonk, NY, USA)] with a two-sided significance threshold of α = 0.05. Continuous variables are presented as mean ± standard deviation (SD) when approximately normally distributed and as median with interquartile range otherwise; the Shapiro–Wilk test and visual inspection of Q–Q plots guided this decision. Categorical variables are summarized as counts and percentages. Between-group comparisons of continuous variables used the independent-samples t-test for two groups and one-way analysis of variance (ANOVA) with Tukey post-hoc correction for three or more groups; categorical associations were tested with the Pearson χ^2^ test, or Fisher’s exact test where expected cell counts were below five.

Predictors of high defensive practice were assessed using multivariable binary logistic regression. Candidate covariates were specified a priori on clinical and theoretical grounds (professional role, surgical specialty, direct and vicarious litigation exposure, and consent literacy) rather than selected solely on the basis of bivariate significance screening; bivariate associations are reported for transparency, but no clinically motivated variable was excluded merely because of a non-significant bivariate test. Because age and years of experience were strongly collinear (r = 0.779; Section 3.3), only one member of this pair was eligible for any given adjusted model, and the final binary model was deliberately restricted to five covariates so that the ratio of outcome events (48 participants in the upper defensive-practice tertile) to fitted variables remained close to the conventional benchmark of ten events per variable [31]; the model is accordingly interpreted as exploratory. Results are reported as adjusted odds ratios (aOR) with 95% confidence intervals. To address Objective 3, we additionally fitted a proportional-odds ordinal logistic regression across defensive-practice tertiles, conducted a moderation analysis testing the pre-specified experience × legal-literacy interaction on malpractice anxiety using hierarchical linear regression, and quantified instrument reliability and item performance. Pearson and Spearman coefficients were used for correlation analyses as appropriate, and the proportional-odds assumption was examined with the Brant test. Missing items below the 15% threshold were handled by available-case analysis. The four a priori hypotheses were tested as follows: H1 with the physician–nurse comparison of informed-consent knowledge, H2 with the one-way ANOVA of malpractice anxiety across departments and its Tukey-adjusted post-hoc contrasts, H3 with the multivariable binary and ordinal regression models, and H4 with the hierarchical moderation analysis and its simple-slope decomposition.

## 3. Results

**Figure 1:**
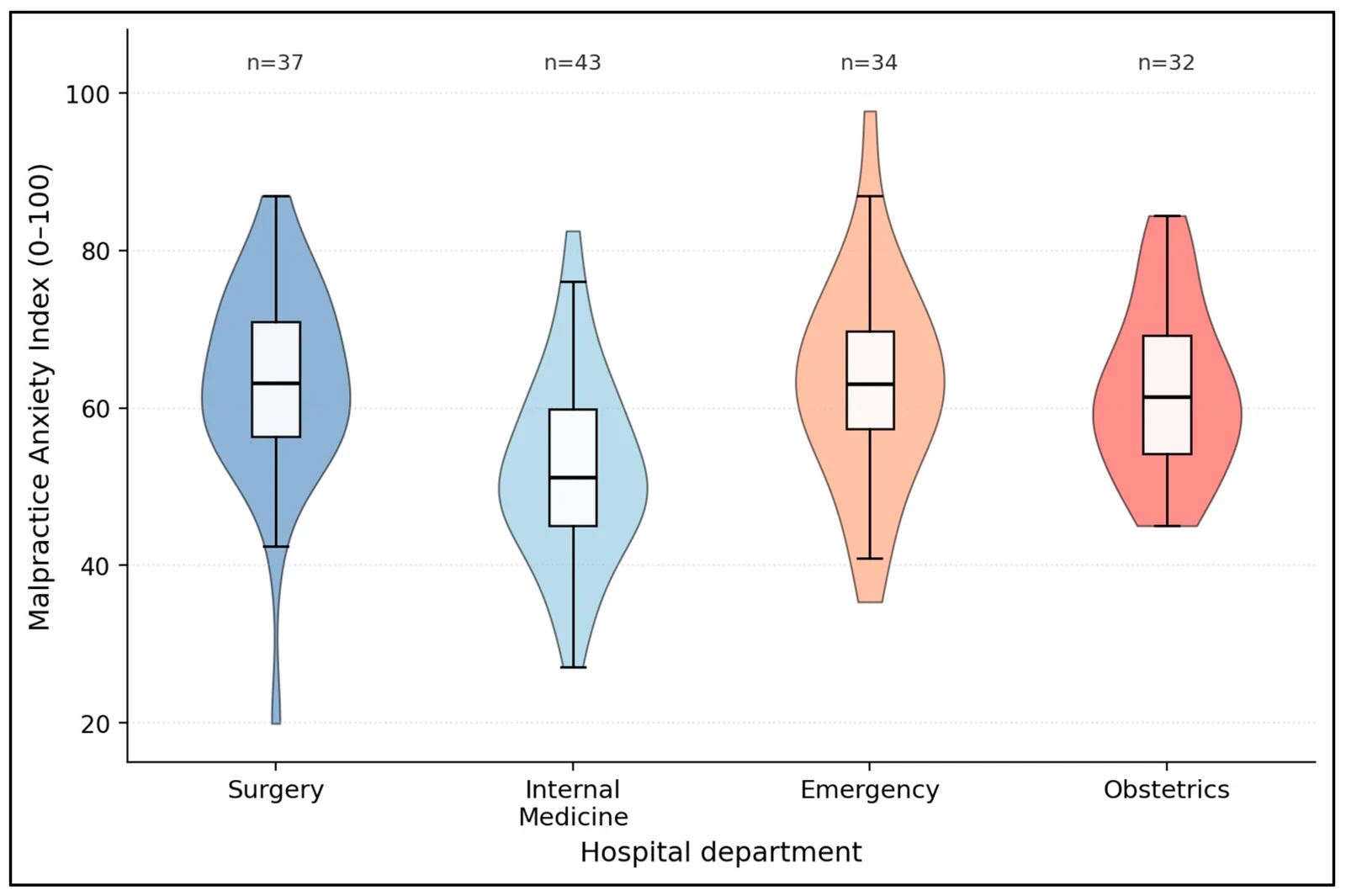
Malpractice Anxiety Index by hospital department. Violin-and-box plots for the four departments (one-way ANOVA, *p* < 0.001); boxes show the median and interquartile range and violins the full kernel-density distribution (*n* = 146). Colors are used only to distinguish the four hospital departments and carry no additional meaning.

**Figure 2:**
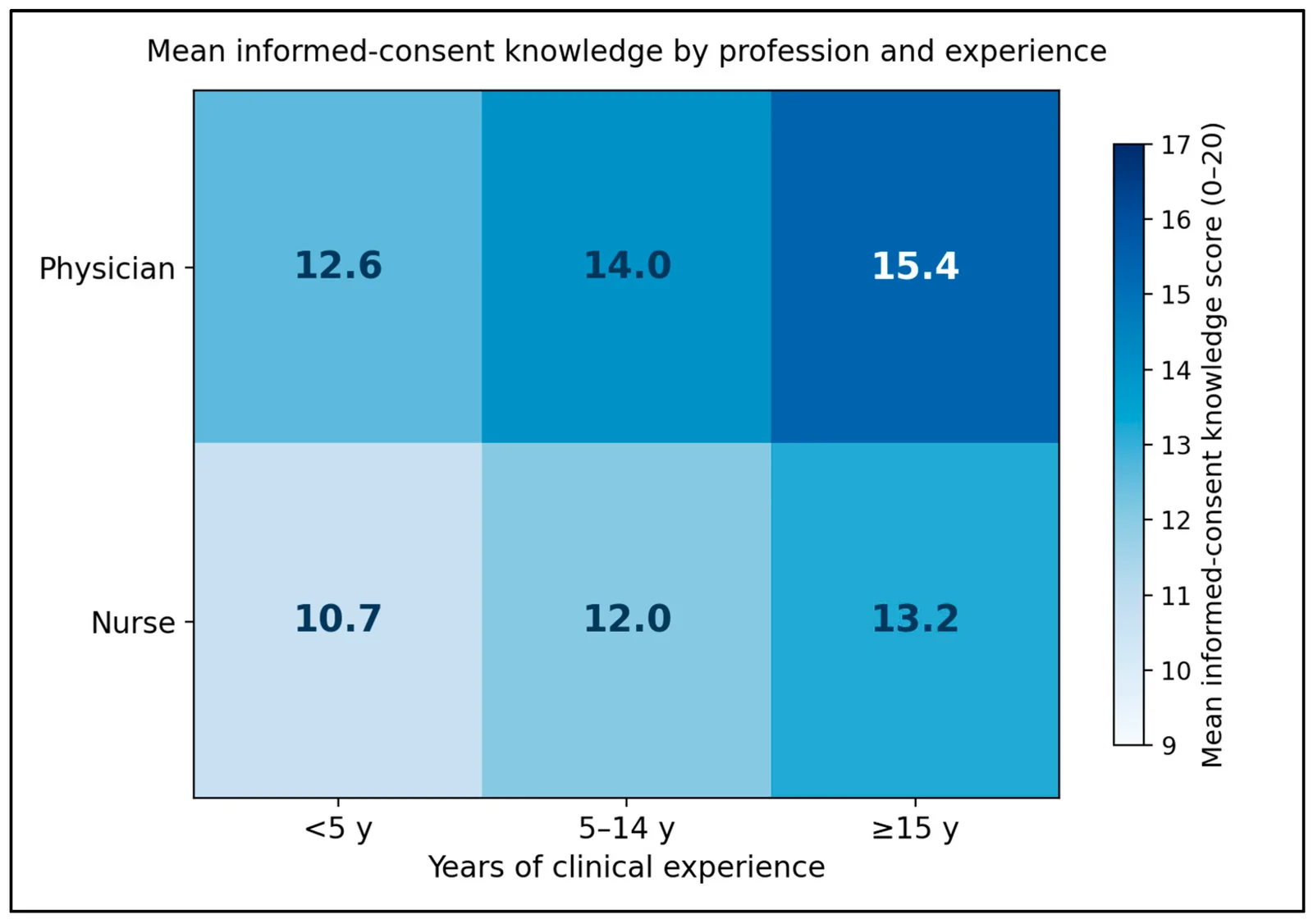
Mean informed-consent knowledge by profession and experience stratum. Heatmap of mean scores (0–20) for physicians and nurses across the three experience strata used throughout the study; darker shades indicate higher scores. Midwives (*n* = 13) and allied health professionals (*n* = 8) are not displayed because their cell sizes would be unstable.

**Figure 3:**
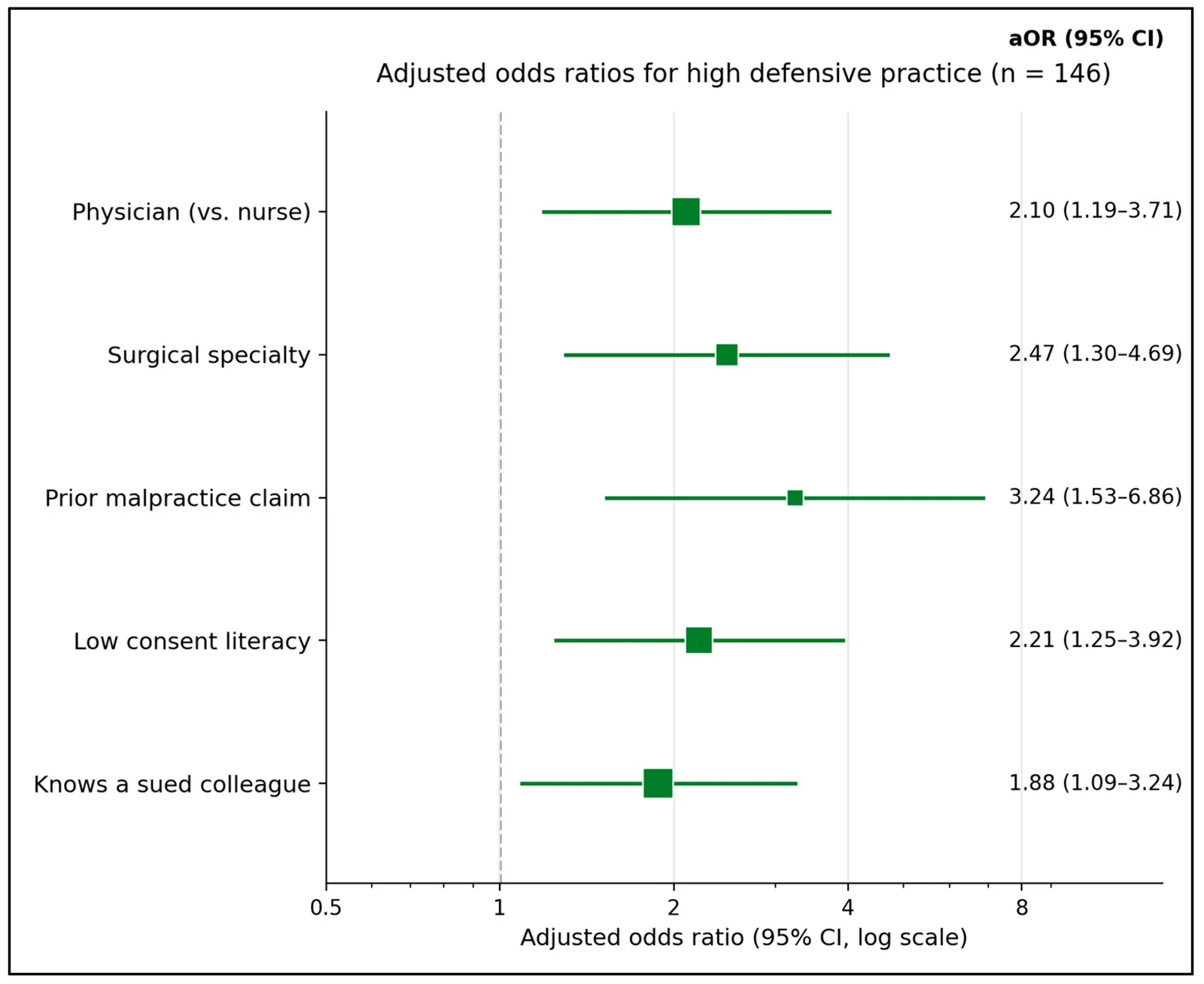
Adjusted odds ratios for high defensive practice. Forest plot of the five-covariate multivariable logistic regression model reported in Table 6 (*n* = 146; log scale); squares denote point estimates with marker size proportional to precision, horizontal lines denote 95% confidence intervals, and the vertical reference line marks the null value (OR = 1.0). A single color is used for all markers and confidence intervals for legibility only and carries no additional meaning; the grey dashed vertical line marks the null value.

A total of 146 healthcare workers were included. The mean age was 38.4 ± 9.6 years, and 95 participants (65.1%) were women. Physicians constituted 47.3% of the sample and nurses 38.4%, with midwives and allied health professionals making up the remainder. The presentation of the results follows the three study objectives: Table 1, Table 2, Table 3 and Table 4 and Figure 1 and Figure 2 address Objective 1; Table 5 and Table 6 and Figure 3 address Objective 2; and Table 7, Table 8 and Table 9 address Objective 3.

### 3.1. Sample Characteristics

The sociodemographic and professional characteristics of the analytic sample are presented in Table 1.

The sample was 65.1% female, and 82.2% of participants were younger than 50 years. Physicians (47.3%) and nurses (38.4%) accounted for 85.7% of respondents, with midwives (8.9%) and allied health professionals (5.5%) forming the remaining strata. Departmental representation ranged from 21.9% (obstetrics) to 29.5% (internal medicine), and 39.7% of participants reported 5–14 years of clinical experience. A prior personal malpractice claim was reported by 15.1% of participants, and 59.6% reported knowing a colleague who had been sued. Most participants worked in the public sector (82.2%).

### 3.2. Distribution of Knowledge, Anxiety, and Defensive Practice Across Groups (Objective 1)

Perception measures were first compared between the two largest professional groups (Table 2).

Physicians scored higher than nurses on informed-consent knowledge (14.1 ± 3.2 vs. 12.0 ± 3.5; t = 3.47, *p* < 0.001) and reported higher malpractice anxiety (63.4 ± 12.9 vs. 57.6 ± 13.2; t = 2.47, *p* = 0.015). None of the categorical contrasts reached significance: high defensive practice (42.0% vs. 28.6%; *p* = 0.119), perceived adequate legal training (31.9% vs. 21.4%; *p* = 0.191), routine documentation of verbal consent (75.4% vs. 62.5%; *p* = 0.120), and reported avoidance of high-risk patients (29.0% vs. 19.6%; *p* = 0.229). Anxiety, knowledge, and defensive practice were then compared across the four clinical departments (Table 3).

Malpractice anxiety differed significantly across departments (F = 10.96, *p* < 0.001), with the highest mean in emergency medicine (67.5 ± 13.4) and the lowest in internal medicine (52.0 ± 11.6); Tukey-adjusted contrasts were significant for emergency versus internal medicine (*p* < 0.001) and for surgery versus internal medicine (*p* = 0.004). Informed-consent knowledge did not differ across departments (range 12.5–13.6; F = 0.67, *p* = 0.573), and neither did the proportion reporting high defensive practice (27.9–50.0%; χ^2^ = 4.66, *p* = 0.199). The corresponding anxiety distributions are shown in Figure 1.

Perception measures were also compared across the three experience strata (Table 4).

Informed-consent knowledge increased across experience strata, from 11.5 ± 3.2 (<5 years) to 14.3 ± 3.1 (≥15 years; Jonckheere–Terpstra *p* < 0.001). Malpractice anxiety also increased, from 55.6 ± 12.5 to 64.0 ± 13.1 (*p* = 0.002), as did the proportion reporting high defensive practice (23.8% to 45.7%; Cochran–Armitage *p* = 0.033) and the proportion knowing a sued colleague (47.6% to 69.6%; *p* = 0.037). Mean knowledge scores for physicians and nurses within each experience stratum are shown in Figure 2: knowledge increased across strata in both professions (physicians: 12.6, 14.0 and 15.4; nurses: 10.7, 12.0 and 13.2), and physicians scored higher than nurses in every stratum.

### 3.3. Correlates and Independent Predictors of High Defensive Practice (Objective 2)

Pearson correlations among the five continuous study variables are presented in Table 5.

Age and experience were strongly correlated (r = 0.779, *p* < 0.001). Informed-consent knowledge correlated with experience (r = 0.221, *p* = 0.007) but not with age (r = 0.108, *p* = 0.194). Malpractice anxiety correlated positively with age (r = 0.232, *p* = 0.005), experience (r = 0.301, *p* < 0.001), and informed-consent knowledge (r = 0.171, *p* = 0.039). The strongest correlation in the matrix was between malpractice anxiety and the defensive-practice score (r = 0.498, *p* < 0.001); informed-consent knowledge was not significantly correlated with defensive practice (r = −0.094, *p* = 0.259). Independent predictors of high defensive practice were then examined in the pre-specified five-covariate logistic regression model (Table 6).

All five covariates were significantly associated with the outcome: prior malpractice claim (aOR 3.24, 95% CI 1.53–6.86, *p* = 0.002), surgical specialty (aOR 2.47, 95% CI 1.30–4.69, *p* = 0.006), low consent literacy (aOR 2.21, 95% CI 1.25–3.92, *p* = 0.007), physician role (aOR 2.10, 95% CI 1.19–3.71, *p* = 0.011) and knowing a sued colleague (aOR 1.88, 95% CI 1.09–3.24, *p* = 0.024). Calibration was adequate (Hosmer–Lemeshow *p* = 0.58), discrimination was moderate (area under the ROC curve 0.75), and the Nagelkerke R^2^ was 0.29. The adjusted odds ratios are displayed in Figure 3.

### 3.4. Ordinal, Moderation, and Reliability Analyses (Objective 3)

To address Objective 3, three additional analyses were conducted: a proportional-odds ordinal regression across defensive-practice tertiles (Table 7), a hierarchical moderation analysis of the pre-specified experience × legal-literacy interaction on anxiety (Table 8), and a psychometric reliability analysis of the instrument (Table 9).

The proportional-odds model was significant overall (likelihood-ratio χ^2^(6) = 41.8, *p* < 0.001; McFadden pseudo-R^2^ = 0.16), and no statistically significant violation of the proportional-odds assumption was detected (all Brant test *p* > 0.05, lowest value 0.087 for low consent literacy). Each 10-point increase in the Malpractice Anxiety Index was associated with 55% higher odds of belonging to a higher defensive-practice category (OR 1.55, 95% CI 1.27–1.89, Wald χ^2^ = 18.7, *p* < 0.001). Prior malpractice claim (OR 2.55, *p* = 0.002), surgical specialty (OR 2.04, *p* = 0.003), low consent literacy (OR 1.86, *p* = 0.008), physician role (OR 1.62, *p* = 0.041) and experience per five years (OR 1.20, *p* = 0.040) were also significantly associated with the outcome.

In Step 1, experience (β = 0.274, *p* < 0.001) and legal literacy (β = 0.158, *p* = 0.038) were positively associated with malpractice anxiety and jointly explained 14.2% of its variance. In Step 2, the experience × legal-literacy interaction was significant (B = −0.031, β = −0.179, t = −2.31, *p* = 0.023) and added 3.1% of explained variance (total R^2^ = 0.173). Simple-slope analysis showed a significant positive association between legal literacy and anxiety at low experience (B = 0.548, t = 2.73, *p* = 0.007) and a non-significant association at high experience (B = 0.088, t = 0.45, *p* = 0.654).

Cronbach’s α ranged from 0.709 for the four-item legal-literacy attitudes subscale to 0.881 for the twelve-item Malpractice Anxiety Index, with corresponding McDonald’s ω values of 0.718 to 0.887. For the full 46-item instrument, α was 0.896, and ω was 0.903, with an average inter-item correlation of 0.179. Mean item–total correlations ranged from 0.408 to 0.554, and the largest gain obtained by deleting the weakest item of a subscale was an increase from 0.709 to 0.732. The Kaiser–Meyer–Olkin measure was 0.81, and Bartlett’s test of sphericity was significant (*p* < 0.001).

## 4. Discussion

### 4.1. Analysis of Findings

This cross-sectional study of 146 hospital healthcare workers yields a coherent and somewhat counter-intuitive portrait of how malpractice liability and informed consent are perceived in contemporary practice [23]. The central finding is that objective legal knowledge and legal anxiety are not inversely related, as a naïve educational model would predict, but instead rise together with profession and experience [32]. Physicians, despite scoring substantially higher on informed-consent knowledge than nurses, reported greater malpractice anxiety and more defensive behavior [33], and the same dissociation reappeared across experience strata and in the correlation matrix [34]. This pattern reframes defensive medicine as a phenomenon driven less by ignorance than by exposure and perceived personal accountability [35]. Two features of the data reinforce this reading. First, informed-consent knowledge was statistically indistinguishable across the four departments, so the large departmental differences in anxiety cannot be attributed to differential legal knowledge. Second, malpractice anxiety was by far the strongest continuous correlate of the defensive-practice score, accounting for approximately a quarter of its variance, whereas knowledge was, if anything, weakly and non-significantly related to defensive practice in the opposite direction. It should be noted that several physician–nurse contrasts did not reach statistical significance; these categorical comparisons were consistent in direction with the continuous effects but underpowered at this sample size and are therefore hypothesis-generating. One of them—the more frequent documentation of verbal consent by physicians—describes an adaptive rather than a maladaptive response to perceived legal risk.

The multivariable and ordinal models converge on a consistent hierarchy of drivers. Prior malpractice claims, surgical specialty, and malpractice anxiety dominated the prediction of defensive practice [36], while low consent literacy remained an independent—and, importantly, modifiable—contributor [37]. The fact that knowing a sued colleague independently predicted defensive practice underscores the social transmission of medico-legal fear: litigation reverberates through a department well beyond the individuals directly involved [38]. Comparable contagion effects of litigation stress have been described in other high-liability specialties [39]. The ordinal model’s satisfaction of the proportional-odds assumption further indicates that these influences operate uniformly across the severity continuum rather than only at the extreme [40], strengthening the case that anxiety is the principal modifiable lever for intervention across the whole workforce [41].

Perhaps the most novel contribution is the moderation analysis, which showed that the positive association between legal literacy and malpractice anxiety was concentrated among healthcare workers with lower clinical experience and was not evident at higher experience [42]. This interaction has practical weight: medico-legal curricula that simply increase awareness of liability may inadvertently heighten anxiety in less-experienced healthcare workers unless paired with coping resources, mentorship, and institutional reassurance [43]. The departmental findings reinforce this targeting logic, localizing the highest anxiety and defensiveness to emergency and surgical settings [9]. Together, the results argue for stratified, context-sensitive interventions rather than uniform legal education, and they position the affective dimension of malpractice perception as a legitimate target for institutional quality improvement [44].

Taken together, and returning to the three study objectives and the four a priori hypotheses, the findings can be synthesized as follows. H1 was supported: physicians scored higher than nurses on informed-consent knowledge. H2 was partially supported: the overall departmental difference in malpractice anxiety was significant, and anxiety was highest in emergency medicine, but the post-hoc contrasts reached significance only for emergency medicine and surgery versus internal medicine. H3 was supported: direct and vicarious litigation exposure and low consent literacy were each independently associated with high defensive practice. H4 was supported: the association between legal literacy and malpractice anxiety was moderated by clinical experience. With respect to Objective 1, knowledge, anxiety, and defensive practice were unevenly distributed across the workforce: physicians and more experienced staff knew more but also worried more, and anxiety clustered in emergency medicine, surgery, and obstetrics. With respect to Objective 2, high defensive practice was independently associated with a prior malpractice claim, surgical specialty, low consent literacy, physician role, and knowing a sued colleague. With respect to Objective 3, the ordinal model indicated that these factors operate across the whole severity range of defensive behavior, and the moderation analysis showed that legal literacy is associated with higher anxiety mainly among healthcare workers at lower levels of clinical experience. Several complementary explanations are plausible. Greater knowledge may sharpen the perception of legal exposure before clinicians have accumulated the reassurance of uneventful practice; senior and procedural staff carry a larger cumulative direct and vicarious “litigation history”; and departmental climates may normalize defensive routines, transmitting them socially in the same way that awareness of sued colleagues appears to transmit anxiety. Alternative explanations cannot be excluded in a cross-sectional design—for example, more anxious clinicians may seek out more legal information, reversing the assumed direction of the literacy–anxiety association.

### 4.2. Study Limitations

Several limitations temper the interpretation of these findings. First, the cross-sectional design precludes causal inference; although the moderation and predictive models are theoretically motivated, the temporal ordering of anxiety, knowledge, and defensive behavior cannot be established, and reverse or reciprocal causation remains possible. Second, all measures were self-reported, raising the possibility of social-desirability bias, particularly for sensitive items concerning the avoidance of high-risk patients; defensive practice was not corroborated against objective utilization data such as imaging or referral rates. Third, the study was conducted within a single academic hospital network in one regional and legal jurisdiction, which may limit generalizability to other medico-legal systems with different liability regimes, insurance structures, or consent norms; in addition, although participants were randomly selected within strata, voluntary participation and the fixed proportional allocation may have introduced residual selection bias. Fourth, despite a strong 85.9% response rate, the modest overall sample of 146 participants limits statistical power: several bivariate categorical contrasts that pointed in the expected direction did not reach significance; the smallest subgroups (midwives and allied health professionals, together fewer than 25 respondents) yield unstable estimates and were therefore not analyzed as separate subgroups; and, even after restricting the binary model to five covariates, the events-per-variable ratio (≈9.6) remained marginally below the conventional benchmark of ten [31], so the adjusted estimates carry wide confidence intervals and should be regarded as exploratory and in need of confirmation in a larger, adequately powered cohort. Fifth, the pre-specified categorization of continuous scores (defensive-practice tertiles and the median split of consent knowledge) facilitates interpretation and comparison with previous surveys but discards information and imposes boundaries on an underlying continuum; the continuous and ordinal analyses were reported in parallel to mitigate this loss, and the borderline Brant test value for low consent literacy warrants cautious reading of the ordinal model. Sixth, the Malpractice Anxiety Index aggregates Likert-type items into a summated score treated as approximately continuous; although this is standard practice for multi-item scales [29,30], the strict interval properties of the underlying responses cannot be guaranteed, and non-parametric sensitivity analyses were therefore reported. Finally, the instrument, although internally reliable and pilot-tested, was newly developed for this study, and its construct validity should be examined by confirmatory factor analysis in an independent sample. Future longitudinal and multi-center studies linking perception to objective practice patterns are warranted.

### 4.3. Implications for Healthcare Practice and Policy

The findings of this study, if confirmed in larger multi-center samples, would have practical relevance for healthcare delivery, patient safety, and health-system governance. Defensive medicine is not merely an individual coping response but a systemic driver of low-value care: assurance behaviors such as the over-ordering of tests, imaging, and referrals translate into avoidable resource utilization, longer patient pathways, and exposure of patients to the iatrogenic risks of unnecessary investigation [45]. Within the limits of a single-network cross-sectional survey, the clustering of anxiety and defensiveness observed in emergency medicine, surgery, and obstetrics suggests that these high-acuity services are a natural starting point for targeted institutional interventions, and that reducing maladaptive defensive practice can be framed as a quality-improvement objective and not only a medico-legal one.

At the level of patient care, the observation that informed-consent literacy is modifiable yet decoupled from reduced anxiety suggests that structured, standardized consent protocols and decision-support tools deserve consideration as part of routine clinical workflows. Such protocols could simultaneously strengthen patient autonomy and shared decision-making—core healthcare-quality goals—while relieving clinicians of the uncertainty that fuels defensive behavior [46]. Because nurses, midwives, and allied health professionals participate substantially in disclosure and consent yet scored lower on consent knowledge than physicians, interprofessional consent training and clearly delineated team roles appear to be practical levers for improving the safety and consistency of the consent process across the care team.

At the workforce level, the finding that malpractice anxiety rises with seniority and is socially transmitted through awareness of sued colleagues suggests that litigation-related distress can also be understood as an occupational-health and clinician-wellbeing issue with direct relevance to burnout, retention, and the sustainability of the health workforce [47]. These findings suggest that medico-legal education may be more effective when paired with peer-support programs, mentorship, and structured second-victim resources around adverse events and complaints, rather than being delivered in isolation. The moderation result—that awareness-raising can be transiently anxiogenic for healthcare workers with lower clinical experience—further suggests that the sequencing and framing of such education should be calibrated to career stage so that competency-building and psychological safety advance together.

For health-system managers and policymakers, these exploratory results are consistent with a shift from reactive, individual-blame responses toward proactive, organization-level strategies that integrate medico-legal risk management with clinical governance, patient-safety reporting, and just-culture frameworks [48]. Should the present associations be replicated in larger, multi-center studies, monitoring the affective climate of departments—and not only their adherence to protocol—could become a useful component of healthcare quality dashboards, helping to direct resources to the services where defensive practice is concentrated [49]. In this way, attending to how clinicians feel about legal risk may become an actionable component of safer, more efficient, and more patient-centered healthcare delivery.

## 5. Conclusions

Within the limits of a single-network cross-sectional survey of 146 hospital healthcare workers, this study addressed its three objectives as follows. First, informed-consent knowledge, malpractice anxiety, and defensive practice were unevenly distributed across the workforce: physicians scored higher than nurses on consent knowledge yet reported greater anxiety, both measures rose with clinical experience, and anxiety was highest in emergency medicine. Second, high defensive practice was independently associated with a prior malpractice claim, surgical specialty, low consent literacy, physician role, and knowing a sued colleague, indicating that direct and vicarious litigation exposure and a modifiable literacy deficit—rather than legal knowledge alone—were the main correlates of defensive behavior. Third, the ordinal model suggested that these factors operate across the whole severity range of defensive practice, and the moderation analysis indicated that greater legal literacy was associated with higher anxiety mainly among healthcare workers with lower clinical experience. These findings suggest—without establishing causality—that medico-legal education alone is unlikely to reduce defensive practice, and that combining such education with structured informed-consent procedures and experience-sensitive professional support is a reasonable strategy to be evaluated in future research. Longitudinal, multi-center studies linking these perceptions to objective practice patterns are needed before firm recommendations for healthcare institutions and policymakers can be made.

## Figures and Tables

**Table 1:** Sample characteristics (*n* = 146).

Characteristic	Category	*n*	%
Sex	Female	95	65.1
	Male	51	34.9
Age group	<35 years	61	41.8
	35–49 years	59	40.4
	≥50 years	26	17.8
Profession	Physician	69	47.3
	Nurse	56	38.4
	Midwife	13	8.9
	Allied health	8	5.5
Department	Surgery	37	25.3
	Internal medicine	43	29.5
	Emergency	34	23.3
	Obstetrics	32	21.9
Experience	<5 years	42	28.8
	5–14 years	58	39.7
	≥15 years	46	31.5
Prior malpractice claim	Yes	22	15.1
Knows a sued colleague	Yes	87	59.6
Hospital sector	Public	120	82.2
	Private	26	17.8

Values are counts and column percentages. Percentages may not sum to 100 due to rounding.

**Table 2:** Perception measures in physicians and nurses.

Measure	Physicians (*n* = 69)	Nurses (*n* = 56)	Test	*p*-Value
Informed-consent knowledge (0–20)	14.1 ± 3.2	12.0 ± 3.5	t = 3.47	<0.001
Malpractice anxiety (0–100)	63.4 ± 12.9	57.6 ± 13.2	t = 2.47	0.015
High defensive practice, *n* (%)	29 (42.0)	16 (28.6)	χ^2^ = 2.43	0.119
Perceived adequate legal training, *n* (%)	22 (31.9)	12 (21.4)	χ^2^ = 1.71	0.191
Routinely documents verbal consent, *n* (%)	52 (75.4)	35 (62.5)	χ^2^ = 2.42	0.120
Would avoid high-risk patients, *n* (%)	20 (29.0)	11 (19.6)	χ^2^ = 1.45	0.229

Continuous variables compared with independent-samples *t*-test; categorical variables with Pearson χ^2^. Statistical significance was set at α = 0.05.

**Table 3:** Perception measures by hospital department.

Department	*n*	Anxiety (0–100), Mean ± SD	Knowledge (0–20), Mean ± SD	Defensive Practice, *n* (%)
Surgery	37	62.6 ± 12.9	13.6 ± 3.3	17 (45.9)
Internal medicine	43	52.0 ± 11.6	12.8 ± 3.6	12 (27.9)
Emergency	34	67.5 ± 13.4	13.1 ± 3.1	17 (50.0)
Obstetrics	32	64.0 ± 12.3	12.5 ± 3.4	14 (43.8)
F statistic		F = 10.96	F = 0.67	χ^2^ = 4.66
*p*-value		<0.001	0.573	0.199

Continuous measures compared by one-way ANOVA; departmental defensive-practice rates by χ^2^ test. Tukey post-hoc: emergency > internal medicine (*p* < 0.001), surgery > internal medicine (*p* = 0.004).

**Table 4:** Perception measures by years of clinical experience.

Experience Stratum	Knowledge (0–20)	Anxiety (0–100)	High Defensive Practice, *n* (%)	Knows Sued Colleague, *n* (%)
<5 years (*n* = 42)	11.5 ± 3.2	55.6 ± 12.5	10 (23.8)	20 (47.6)
5–14 years (*n* = 58)	13.0 ± 3.4	60.9 ± 12.8	21 (36.2)	35 (60.3)
≥15 years (*n* = 46)	14.3 ± 3.1	64.0 ± 13.1	21 (45.7)	32 (69.6)
Trend test	*p* < 0.001	*p* = 0.002	*p* = 0.033	*p* = 0.037

Linear trend assessed by Jonckheere–Terpstra test (continuous) and Cochran–Armitage trend test (proportions).

**Table 5:** Correlation matrix of continuous study variables.

Variable	(1)	(2)	(3)	(4)	(5)
(1) Age	—	<0.001	0.194	0.005	0.146
(2) Experience (years)	0.779	—	0.007	<0.001	0.094
(3) IC knowledge	0.108	0.221	—	0.039	0.259
(4) Malpractice anxiety	0.232	0.301	0.171	—	<0.001
(5) Defensive-practice score	0.121	0.139	−0.094	0.498	—

Coefficients below the diagonal are Pearson’s r; values above the diagonal are corresponding two-tailed *p*-values. *n* = 146.

**Table 6:** Logistic regression for high defensive practice.

Predictor	β	aOR	95% CI	*p*-Value
Physician (vs. nurse)	0.742	2.10	1.19–3.71	0.011
Surgical specialty	0.904	2.47	1.30–4.69	0.006
Prior malpractice claim	1.176	3.24	1.53–6.86	0.002
Low consent literacy	0.793	2.21	1.25–3.92	0.007
Knows a sued colleague	0.631	1.88	1.09–3.24	0.024

Model restricted a priori to five covariates (events-per-variable ≈ 9.6; 48 outcome events). Hosmer–Lemeshow goodness-of-fit *p* = 0.58; Nagelkerke R^2^ = 0.29; area under ROC curve = 0.75. Reference categories: nurse, non-surgical specialty, adequate literacy, no prior claim, does not know a sued colleague.

**Table 7:** Ordinal regression across defensive-practice tertiles.

Predictor	Ordinal OR	95% CI	Wald χ^2^	*p*-Value	Brant Test *p*
Malpractice anxiety (per 10 pts)	1.55	1.27–1.89	18.7	<0.001	0.198
Surgical specialty	2.04	1.27–3.28	8.7	0.003	0.341
Prior malpractice claim	2.55	1.40–4.65	9.3	0.002	0.176
Low consent literacy	1.86	1.18–2.93	7.2	0.008	0.087
Physician (vs. nurse)	1.62	1.02–2.57	4.2	0.041	0.402
Experience (per 5 years)	1.20	1.01–1.43	4.2	0.040	0.149

Proportional-odds model with three ordered outcome levels. Overall model likelihood-ratio χ^2^(6) = 41.8, *p* < 0.001; pseudo-R^2^ (McFadden) = 0.16. All Brant test *p*-values > 0.05, supporting the proportional-odds assumption.

**Table 8:** Moderation of the experience × legal-literacy interaction on anxiety.

Model Term	B (SE)	β	t	*p*-Value	ΔR^2^
Step 1—Main effects					0.142
Experience (centered)	0.398 (0.118)	0.274	3.37	<0.001	
Legal literacy (centered)	0.305 (0.146)	0.158	2.09	0.038	
Step 2—Interaction					0.031
Experience × Legal literacy	−0.031 (0.013)	−0.179	−2.31	0.023	
Simple slope: low experience	0.548 (0.201)		2.73	0.007	
Simple slope: high experience	0.088 (0.196)		0.45	0.654	

Hierarchical linear regression; dependent variable = Malpractice Anxiety Index. Variables mean-centered prior to forming the product term. Total model R^2^ = 0.173, F(3, 142) = 9.90, *p* < 0.001. Simple slopes evaluated at ±1 SD of experience.

**Table 9:** Reliability and item performance of the instrument.

Subscale	Items	Cronbach’s α	McDonald’s ω	Mean Item–Total r	α If Worst Item Dropped
Informed-consent knowledge	20	0.836	0.844	0.408	0.843
Malpractice anxiety	12	0.881	0.887	0.554	0.886
Defensive practice	10	0.784	0.796	0.421	0.803
Legal-literacy attitudes	4	0.709	0.718	0.461	0.732
Full instrument	46	0.896	0.903	0.179	—

Reliability estimated on the full analytic sample (*n* = 146). Average inter-item correlation for the full instrument = 0.179; Kaiser–Meyer–Olkin measure of sampling adequacy = 0.81; Bartlett’s test of sphericity *p* < 0.001.

## Data Availability

The data presented in this study, together with the verbatim questionnaire and item-level scoring key, are available from the corresponding author upon reasonable request; participant-level data are not publicly available due to privacy and ethical restrictions.

## Appendix A. Supplementary Scoring Guide for the Study Instrument

The instrument contains 46 scored items distributed across four subscales. The informed-consent knowledge scale contains 20 dichotomously scored items; each item contributes 0 or 1 point, yielding a total from 0 to 20, with higher scores indicating greater objective knowledge. For analyses requiring a binary literacy indicator, low versus adequate consent literacy was defined using the pre-specified sample-median threshold described in Section 2.3.

The Malpractice Anxiety Index contains 12 items rated from 1 to 5. The raw sum ranges from 12 to 60 and was linearly transformed to a 0–100 scale using 100 × (raw sum − 12)/48, with higher values indicating greater anxiety.

The defensive-practice subscale contains 10 items rated from 1 (never) to 5 (always), yielding a total from 10 to 50. The pre-specified tertile categories used for regression were low (10–24), moderate (25–31), and high (≥32); continuous-score and ordinal analyses were reported in parallel to reduce information loss from categorization.

The Legal-Literacy Attitudes subscale contains 4 items and was used descriptively and in reliability analyses. Internal-consistency statistics for all subscales and the full instrument are reported in Table 9. The verbatim questionnaire is not reproduced in this appendix; the original item wording and item-level coding key are available from the corresponding author.
